# Perceptual constancy for an odor is acquired through changes in primary sensory neurons

**DOI:** 10.1126/sciadv.ado9205

**Published:** 2024-12-11

**Authors:** Mark Conway, Merve Oncul, Kate Allen, Zongqian Zhang, Jamie Johnston

**Affiliations:** School of Biomedical Sciences, Faculty of Biological Sciences, University of Leeds, Leeds, UK.

## Abstract

The ability to consistently recognize an object despite variable sensory input is termed perceptual constancy. This ability is not innate; rather, it develops with experience early in life. We show that, when mice are naïve to an odor object, perceptual constancy is absent across increasing concentrations. The perceptual change coincides with a rapid reduction in activity from a single olfactory receptor channel that is most sensitive to the odor. This drop in activity is not a property of circuit interactions within the olfactory bulb; instead, it is due to a sensitivity mismatch of olfactory receptor neurons within the nose. We show that, after forming an association of this odor with food, the sensitivity of the receptor channel is matched to the odor object, preventing transmission failure and promoting perceptual stability. These data show that plasticity of the primary sensory organ enables learning of perceptual constancy.

## Teaser

Learning-induced changes in primary sensory neurons support concentration-invariant perception of an odor.

## INTRODUCTION

Perceptual constancy is fundamental to experiencing the world; it enables consistent recognition of an object despite sensory input that may vary depending on lighting conditions (*1*, *2*), pitch and loudness (*3*), or speed of touching an object (*4*). This ability does not appear to be innate; rather, it is thought to develop with experience (*1*, *2*, *5*). Identifying the neural changes that coincide with the development of perceptual constancy poses a considerable challenge, given that interaction with objects is difficult to restrict for most sensory modalities. We have taken advantage of the olfactory system of mice, where, in a laboratory setting, experience of odors is intrinsically restricted, allowing us to compare experience-induced changes in perception. Perceptual constancy for an odor can be affected by at least two factors: the concentration of the odor, which will vary with distance to the source, and the presence of a background odor. Previous works have demonstrated that mice can learn to identify a target odor despite the presence of temporally coherent background odors (*6*) and can also learn to separate odor sources based on temporal incoherence (*7*). Here, we examine how mice perceive a naïve odor across a range of concentrations and show that the perception of this odor changes after it has been associated with food and that this shift corresponds to changes in how odor information is delivered to the olfactory bulb.

## RESULTS

### Shifts in odor perception occur with concentration

**Fig. 1.**
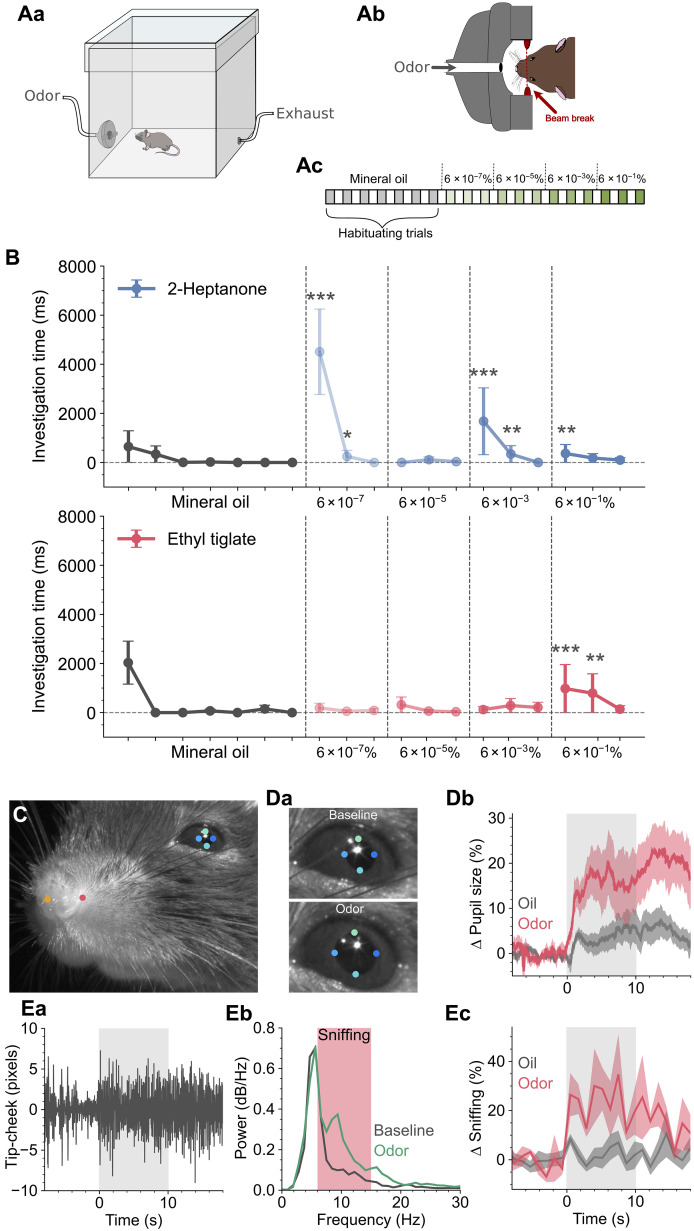
Concentration-dependent changes in olfactory perception. (**Aa**) Experimental paradigm; mice were placed in a test chamber with an odor delivery port and exhaust. (**Ab**) The odor delivery port contained a nose poke with a beam break sensor to log investigation times. (**Ac**) Odor delivery protocol; each block represents 60 s (60-s stimulus, 60-s interstimulus interval). (**B**) Odor investigation times during stimulus delivery for 2-heptanone and ethyl tiglate; data are displayed as median ± the median absolute deviation, *N* = 32. The horizontal dashed line indicates the basal amount of investigation calculated from the last five oil presentations. There were significant differences between the last five oil presentations and the odor presentations for both 2-heptanone and ethyl tiglate (*P* = 1.15 × 10^−20^ and *P* = 1.7 × 10^−5^, Friedman test). Asterisks indicate post hoc significance tests compared to basal investigation (see Materials and Methods). (**C**) Mice were head-fixed and facial features were tracked with DeepLabCut (see Materials and Methods); colored dots indicate key points tracked. (**Da**) Pupil diameter before and during odor stimulation; diameter was calculated as the mean from the cardinal points. (**Db**) Relative change in pupil diameter displayed as means ± SEM during presentation of 1 × 10^−7^% ethyl tiglate (red) and for three preceding stimulus blanks (gray), *N* = 6; stimulus period indicated by gray shaded area. (**Ea**) Oscillations in the distance between the key points for the nose tip and cheek. (**Eb**) Fourier transforms of the data in (Ea), for 10 s before stimuli (gray) and during stimulation (green); sniffing band from (*9*) indicated by red shaded box. (**Ec**) Change in the mean power of the sniffing band displayed as mean ± SEM during presentation of 1 × 10^−7^% ethyl tiglate (red) and for three preceding stimulus blanks (gray), *N* = 6; stimulus period indicated by gray shaded area.

To evaluate whether mice experience a perceptual change in response to varying concentrations of an odorant, we used a cross-habituation assay, a standard method used to determine a rodent’s ability to differentiate between odorants (*8*–*11*). We used an automated approach based on (*10*), where mice were placed in a test chamber with odors delivered through a nose-poke containing a beam break that logged investigation time (Fig. 1A). Cross-habituation assays rely on two criteria being met: (i) the mouse can detect the odor and (ii) the mouse is motivated to investigate the odor. We therefore started with 2-heptanone, a component of mouse urine (*12*), with the rationale that mice should investigate this odor if detected. Mice investigated 2-heptanone at the lowest concentration tested (6 × 10^−7^%) and then rapidly habituated to two subsequent presentations; this habituated state was maintained even with a 100-fold jump in concentration to 6 × 10^−5^% (Fig. 1B). When the concentration was increased 10,000 times above that of the original, the mice once more investigated the odor with a similar pattern of habituation to further stimuli (Fig. 1B). This indicates that mice perceived a qualitative change in the odor between 6 × 10^−5^ and 6 × 10^−3^% but did not between the two lowest concentrations. We next used ethyl tiglate, an odor to which the mice were naïve. In this case, the mice failed to investigate for all concentrations up until 6 × 10^−1^% (Fig. 1B). Rather than the mice perceiving a qualitative difference between the concentrations of 6 × 10^−3^ and 6 × 10^−1^%, the failure of the mice to investigate at lower concentrations could merely reflect their inability to detect these concentrations. We therefore developed a method to measure the detection ability of mice to novel odors that is independent of internal motivation. We head-fixed the mice on a treadmill (*13*) and, with video recording, tracked key facial features with DeepLabCut (*14*) (Fig. 1C). In both humans and rodents, detection of a novel stimulus results in pupil dilation (*15*, *16*) and we find that 1 × 10^−7^% of ethyl tiglate results in significantly larger pupil dilation than preceding blanks containing only the solvent (Fig. 1D). In addition, we tracked key points around the snout and noticed that the nose tip moves relative to the cheek seemingly in phase with breathing. When we plotted the distance between these key points (Fig. 1Ea), we found oscillations around resting respiration frequencies of ~2 to 5 Hz (*9*). Notably, during stimulation with ethyl tiglate at 1 × 10^−7^%, there was a significant increase in the frequency content linked to sniffing/active exploration (Fig. 1, Ea and Eb; *N* = 6). These data demonstrate that mice can detect lower concentrations of ethyl tiglate than were delivered in the cross-habituation experiments in Fig. 1B, yet they do not investigate even when the concentration is 60,000 times higher. This is consistent with mice perceiving esters, such as ethyl tiglate, as having a neutral valence (*17*); however, when the concentration reaches 6 × 10^−1^% the mice begin to investigate, indicating that the mice have perceived a qualitative change in the odor.

Together, these data indicate that mice can detect both 2-heptanone and ethyl tiglate at the lowest concentrations tested and that, with increasing concentrations, a perceptual shift occurs, between 6 × 10^−5^ and 6 × 10^−3^% for 2-heptanone and between 6 × 10^−3^ and 6 × 10^−1^% for ethyl tiglate. We next sought to determine the neural basis for generating distinct percepts of the same molecule at different concentrations.

### Odor percepts rely on a sparse code

**Fig. 2.**
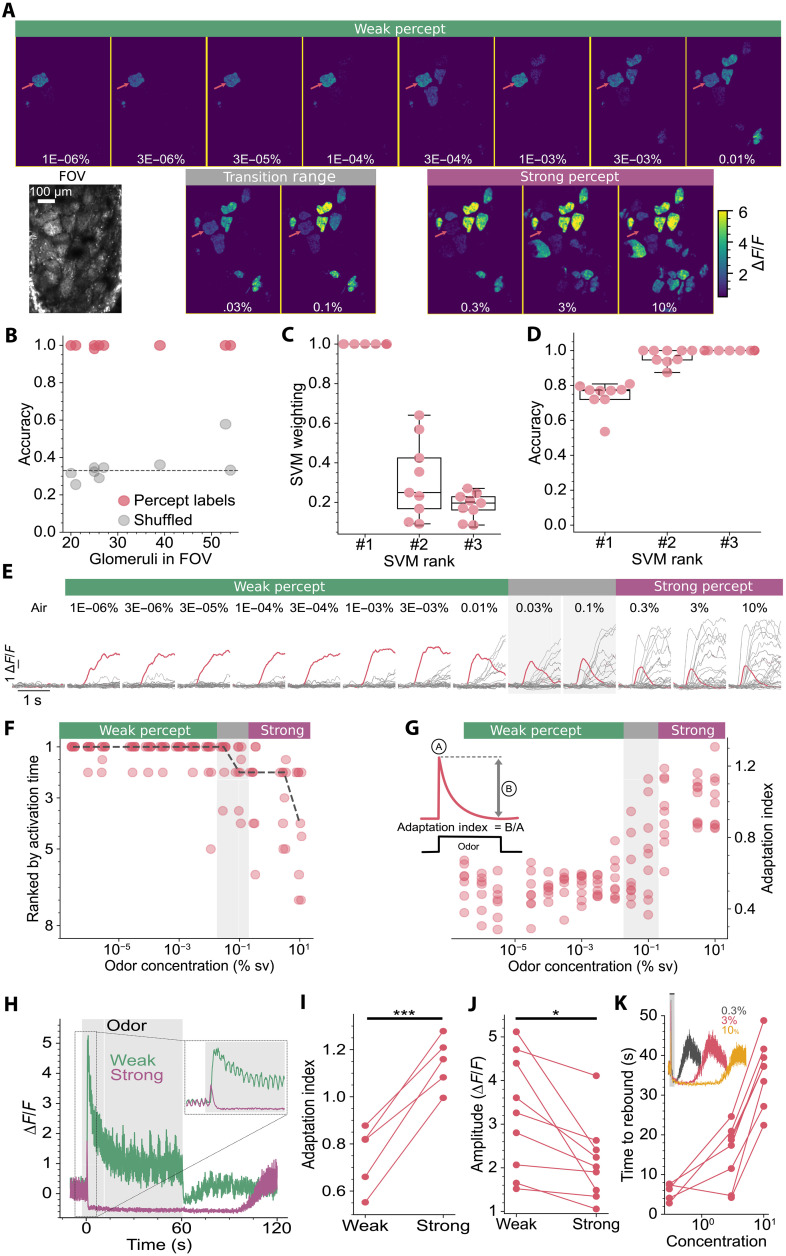
Neural correlates of perceptual shifts. (**A**) Response maps and field of view (FOV) in a Pcdh21xGCaMP6f mouse, showing the mean activity during 3-s odor stimuli for concentrations indicated in white and grouped by odor percept (see text). Red arrows, primary glomerulus. (**B**) Support vector machine (SVM) classifier performance using response maps from nine mice (red dots) and performance with shuffled labels (gray dots). (**C**) Relative classifier weights for the top 3 glomeruli normalized to the primary glomerulus. (**D**) Classifier performance using the top 3 glomeruli identified in (C), *N* = 9. (**E**) Responses of all glomeruli in (A) to single odor trials, primary glomerulus in red, sampled at 42 Hz. Odor percepts indicated with colored bars. (**F**) Activation rank of the primary glomerulus across concentration, each mouse represented by a dot at each concentration, with jitter added for clarity. Black dashed line is median, *N* = 9. (**G**) AI of the primary glomerulus with concentration, each mouse represented by a dot at each concentration; inset shows the calculation of AI, *N* = 9. (**H**) Response of a primary glomerulus to a 60-s stimulus of ethyl tiglate from the weak (1 × 10^−6^%) and strong percepts (3%). Inset: Expanded view of initial response. Note the delayed rebound in activity long after the stimulus ends. (**I**) Adaptation indices of primary glomeruli to a 60-s stimulus were significantly larger for the strong percept (*P* = 0.0008, paired *t* test), *N* = 5. (**J**) Response amplitudes from the primary glomerulus for 3-s odor stimuli were larger for the weak (3 × 10^−3^%) than for the strong percept (10%) (*P* = 0.004, Wilcoxon-signed rank test), *N* = 9. (**K**) The delay to rebound in the primary glomerulus increases with stimulus strength, calculated from 3-s stimuli, *N* = 8; inset: example glomerulus; gray bar indicates the 3-s stimulus.

To explore how the brain represents the range of concentrations used in Fig. 1, we used in vivo two-photon imaging. We used the genetically encoded Ca^2+^ indicator GCaMP6f (*18*) expressed in mitral and tufted cells of the olfactory bulb, driven by the Pcdh21 promoter (*19*) (Pcdh21xGCaMP6f mice; see Materials and Methods). We began by imaging the odor-evoked responses in the glomerular layer, the site of the initial excitation of these output neurons. This approach enables visualization of the spatiotemporal activity arriving in the olfactory bulb (*20*) as each glomerulus corresponds to input from a single olfactory receptor (*21*). We presented mice with concentrations of ethyl tiglate spanning the entire range used in the cross-habituation experiments (Fig. 1). We generated response maps (Fig. 2A) by averaging glomerular activity over the 3-s stimulus period. As in the cross-habituation experiments, mice were presented with the most dilute concentration first, with each successive stimulus 3- to 10-fold stronger. Glomerular responses were detected at every concentration presented, supporting the finding that mice can detect ethyl tiglate over six orders of magnitude (Fig. 1). In accordance with a recent report (*22*), glomerular responses to the weakest concentrations were sparse, with generally only a single glomerulus responding to most concentrations presented from the weak percept (Fig. 2A). As expected, the total number of active glomeruli was far greater when mice were presented with higher concentrations of the same odor (*23*, *24*). We assigned labels to the responses based on the cross-habituation experiment; responses between the weakest stimuli and 0.01% were labeled as the “weak percept,” and responses above 0.3% were labeled as the “strong percept,” which includes concentrations within 50% of the boundaries of the perceptual shift. We did not identify the precise concentration where the perceptual shift occurs, which may vary depending on nasal patency, but it falls between ~6 × 10^−3^ and ~6 × 10^−1^%, which we have termed the “transition range” (Fig. 2A).

Notably, a linear classifier had a 99.8% success rate in predicting the odor percept based on the neural activity (Fig. 2B, *N* = 9). As the performance did not seem to depend on the number of glomeruli in the field of view, we next examined the weights assigned to each glomerulus used in the classifier; these weights directly signify the extent to which each glomerulus contributes to the decision boundary. We found that a single glomerulus in each mouse made a major contribution, with the second and third most important having weights of 25 ± 15% and 19 ± 3% of the first (Fig. 2C). Unexpectedly, when we used only the single most important glomerulus, the classifier achieved 74% accuracy, and with only two glomeruli, this increased to 96.7%, a comparable performance to using all glomeruli (Fig. 2D). This suggests that only a few glomeruli are necessary to encode the odor percepts rather than a broad pattern of active glomeruli.

Previous works have indicated that a sparse “primacy” code may be used for odor identity (*25*, *26*), whereby the fastest activating glomeruli carry the most importance. Our data are consistent with such a primacy code; when glomeruli were ranked in the order they activated, we found that the glomerulus with the strongest predictive value was also the glomerulus that activated first (Fig. 2, E and F). However, this was only true for the weaker percept; for the strong percept, this glomerulus began to lag other glomeruli that became active at higher concentrations (Fig. 2, E and F). Nevertheless, as this glomerulus contributed most to classifying the odor percept and was the first to activate for the weak percept, we will refer to it as the “primary” glomerulus. The most notable behavior of primary glomeruli was that they shift from a sustained response in the weak percept to rapid adaptation for the strong percept. We used the adaptation index (AI) (Fig. 2G) to quantify the amount of adaptation as a function of concentration. An AI of 1 indicates complete adaptation, whereas greater than 1 corresponds to adaptation that reduces the response to below baseline. As can be seen in Fig. 2G, the amount of adaption of the primary glomerulus shifts from 0.34 ± 0.03 for the highest concentration of the weak percept to near-complete adaptation at the higher concentrations with an AI of 1 ± 0.05 for the strong percept (*P* = 1.18 × 10^−7^, paired *t* test, *N* = 9) and this shift to near-complete adaptation occurs within the transition range (Fig. 2G). Together, these data indicate that odor percepts are likely generated using a sparse code, requiring just a few glomeruli, and that a change in perception corresponds to rapid adaptation of the primary glomerulus.

The difference in how the primary glomerulus responds to weak and strong percepts becomes especially evident when 60-s stimuli are delivered (Fig. 2H), mirroring the duration used in the cross-habituation experiments of Fig. 1. Weak percepts show slow and incomplete adaptation (AI = 0.75 ± 0.05, *N* = 5), continuing to respond all throughout the stimulus, whereas strong percepts generate rapid and complete adaptation. Notably, the response to stronger stimuli falls below baseline with an AI of 1.15 ± 0.04 (Fig. 2, H and I), for the five animals where both stimuli (60 s) were delivered. Two further characteristics are of note when comparing responses to the strong and weak percepts: the peak amplitude was smaller for the stronger percept than the weak (Fig. 2J), and a rebound in activity was observed (1.35 ± 0.28 ∆*F*/*F*, *N* = 9), the delay to which depended on the strength of the stimulus (Fig. 2K). We found similar response dynamics in mitral and tufted cell somas in the three animals where we also imaged in deeper layers.

### Rapid adaptation is due to transmission failure from olfactory receptor neurons

**Fig. 3.**
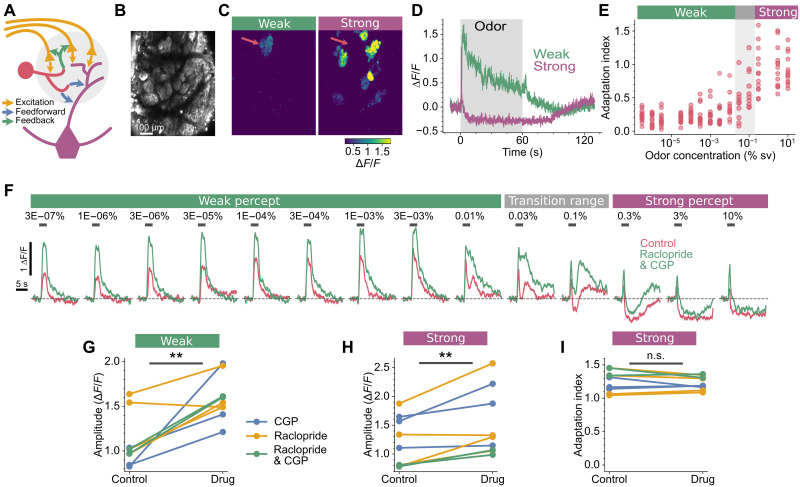
Rapid adaptation does not arise within the olfactory bulb. (**A**) Intraglomerular circuitry within the olfactory bulb. Glutamatergic olfactory receptor neurons (yellow) synapse onto GABAergic periglomerular cells (red), prompting feedback and feedforward inhibition onto olfactory nerve terminals and mitral/tufted cells (purple), respectively. (**B**) Field of view in an OMPxGCaMP6f mouse. (**C**) Response maps for (B), showing the mean activity during 60-s odor stimuli for a weak percept and strong percept (1.0 × 10^−4^ and 3%, respectively). Red arrows indicate the location of the primary glomerulus. (**D**) Time courses of the responses in (C). (**E**) The AI of the primary glomerulus increases when the concentration reaches the strong percept, calculated from 3-s stimuli, *N* = 13 (calculation of AI shown in Fig. 2G), each mouse represented by a dot at each concentration. (**F**) Responses from a primary glomerulus to single odor trials before and after application of the D_2_ and GABA_B_ antagonists raclopride and CGP 54626, respectively. (**G** and **H**) Response amplitudes for the primary glomerulus were larger after drug application (raclopride and/or CGP 54626) at both the weak [(G); *P* = 0.0018, paired *t* test] and strong [(H); *P* = 0.0058, paired *t* test] percepts. Measured from 3-s odor stimuli. Each ball and stick represents an individual mouse, *N* = 9. (**I**) The AI of the primary glomerulus to 60 s of the strong percept was unaffected by drug application (*P* = 0.33, paired *t* test), *N* = 9. n.s., not significant.

In addition to the mitral and tufted output neurons, the olfactory nerve terminals deliver their signal to periglomerular and short axon cells. These juxtaglomerular neurons can provide both feedforward inhibition onto mitral/tufted cells and feedback inhibition onto the olfactory nerve terminals (Fig. 3A). We next tested whether either of these circuit motifs could give rise to rapid adaptation that generates a smaller peak response, a drop in activity below baseline, and a subsequent rebound in activity. Such response dynamics are a hallmark of feedforward inhibition (*27*, *28*). The olfactory nerve input excites both the mitral/tufted dendrites—where the measurements in Fig. 2 are taken—and inhibitory periglomerular neurons. Subsequently, the periglomerular neurons deliver delayed inhibitory drive to the mitral/tufted dendrites (*29*). To test whether such a mechanism gives rise to the fast adaption, we took advantage of mice where GCaMP6f expression is restricted to the olfactory receptor neurons (OMPxGCaMP6f, see Materials and Methods) (*30*, *31*). If feedforward inhibition underpins the observed rapid adaption, then it should only manifest in the mitral/tufted cells not in the olfactory nerve input. We were easily able to identify the same primary glomerulus in OMPxGCaMP6f mice as, across animals, glomeruli are located in almost identical locations (*32*) and, at very low concentrations, glomerular activation is sparse and structured (Figs. 2A and 3C) (*22*). Unexpectedly, the same phenomenon was evident in the olfactory nerve terminals of the primary glomerulus; when we compared 60-s responses between weak and strong percepts, the same switch to rapid adaption was evident (Fig. 3D). The transition between sustained and adapting responses (Fig. 3E) coincides with both the mitral/tufted transition (Fig. 2G) and the perceptual shift (Fig. 1B). As feedforward inhibition does not account for the observed rapid adaptation, we next explored whether feedback inhibition is involved. Sensory input to the olfactory bulb can be modulated by both olfactory bulb activity (*33*) and by centrifugal neuromodulatory systems (*34*–*37*). These all converge to act upon γ-aminobutyric acid type B (GABA_B_) and dopamine D_2_ receptors on the presynaptic terminals of olfactory receptor neurons (*34*, *35*, *37*), reducing presynaptic calcium influx (*38*, *39*). To test whether such presynaptic feedback inhibition could explain the rapid adaptation in the olfactory nerve terminals, we used topical application of CGP 54626 and raclopride, antagonists of GABA_B_ and D_2_ receptors, respectively (*40*, *41*). As expected, disrupting feedback inhibition led to increased presynaptic Ca^2+^ influx for both weak and strong percepts (Fig. 3, F to H), indicating that the drugs were exerting their expected action. However, with feedback inhibition disrupted, the olfactory nerve terminals still displayed the same rapid adaptation to the strong percept (Fig. 3, F and I). These data demonstrate that rapid adaptation in the primary glomerulus, which coincides with a shift in odor percept, is not a feature that is computed by neural circuits in the brain; rather, this signal must already be present in the olfactory receptor neurons located in the nasal epithelium.

**Fig. 4.**
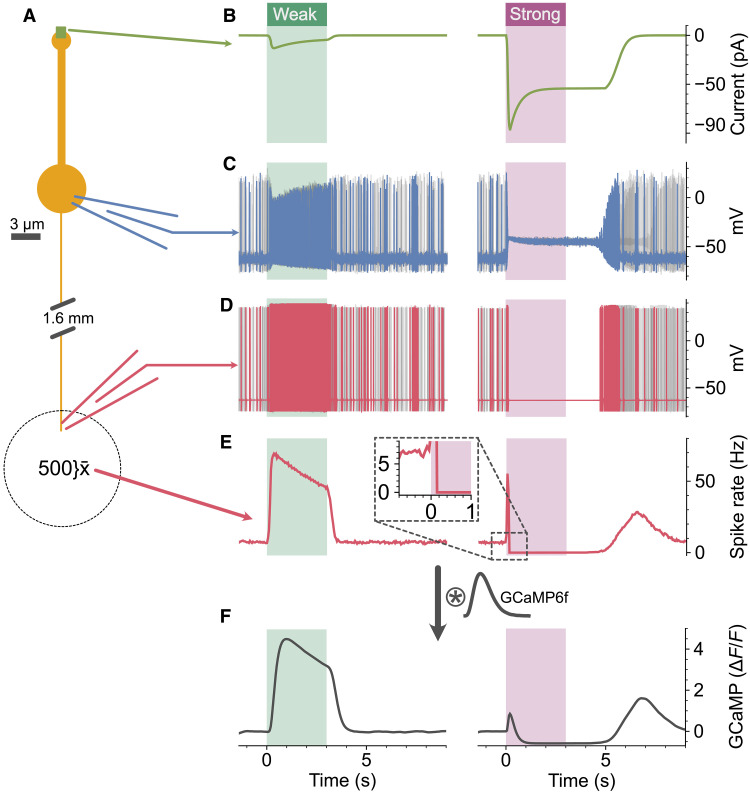
Transmission failure in olfactory receptor neurons results in rapid adaptation within the glomerulus. (**A**) Morphology of the model olfactory receptor neuron. (**B**) Olfactory receptor currents for weak and strong odor stimuli. (**C**) Somatic membrane potential recording for a single neuron in blue for weak and strong stimuli, with four further cells shown in gray. (**D**) Axonal membrane potential recording for a single neuron in red for weak and strong stimuli, with four further cells shown in gray. (**E**) Peristimulus time histograms showing the mean spike rates for 500 simulated neurons. Inset shows the magnified view of the onset for the strong response; note the response falls below baseline. (**F**) Spike rates from (E) convolved with the kinetics of the GCaMP6f reporter. See Materials and Methods for model details.

Adaptation of the olfactory transduction cascade has been well documented (*42*–*44*), where Ca^2+^-dependent feedback reduces sensitivity of the cyclic nucleotide–gated current (*42*). However, it is hard to picture how such a mechanism could give rise to the adaptation that we observe in the olfactory nerve terminals, particularly as a decrease below baseline is observed during the stimulus (Fig. 3, D and I). To understand how this phenomenon arises, we used a morphologically and biophysically realistic model of olfactory receptor neurons (Fig. 4A). The model featured comparable membrane resistances, spontaneous spike rates, and receptor currents as observed in in vitro recordings (Fig. 4, A to F, see Materials and Methods) (*44*, *45*). In the model, we could record the membrane potential from individual olfactory receptor neurons at both the soma and at the olfactory nerve terminals in response to receptor currents corresponding to weak and strong concentrations of odorant (Fig. 4, C and D). However, in our imaging experiments (Figs. 2 and 3), we used a calcium indicator to measure the average activity due to the several thousand olfactory receptor neurons projecting to a glomerulus (*21*). To obtain equivalent recordings in our model, we simulated 500 olfactory receptor neurons (Fig. 4E) and convolved their mean spike rate with the kinetics of the GCaMP6f reporter (Fig. 4F). This model provides important insight into the mechanism of rapid adaptation. The weak stimulus we provided had a peak receptor current of 13 pA, and this resulted in a sustained increase in firing of individual olfactory receptor neurons, which showed slow adaptation at the population level (compare Fig. 4F with Figs. 2, E, F, and H, and 3D). In contrast, the strong stimulus resulted in sustained depolarization at the soma, which generated a few action potentials at the onset of the stimulus that rapidly reduced in amplitude, due to accumulation of voltage-gated Na^+^ (Nav) channels in their inactivated state. The somatic membrane remained in a depolarized state, preventing recovery from inactivation of the Nav channels, thus blocking action potentials from passing down the axons. The resultant population spike rate, when convolved with the kinetics of the GCaMP6f reporter (Fig. 4F), displays all the characteristics reported in Figs. 2 and 3: (i) The brief initial burst of action potentials generates a smaller Ca^2+^ signal than the weaker stimulus, due to the low-pass filtering of the GCaMP6f reporter (Fig. 4F versus Fig. 2, H and J, and Fig. 3D). (ii) The response rapidly drops below the prestimulus baseline, due to the depolarizing block terminating spontaneous action potential firing (Figs. 2H, 3D, and 4, E and F). (iii) A rebound in action potential firing is observed after termination of the stimulus as, once the somatic membrane potential becomes sufficiently hyperpolarized to support recovery from inactivation, the Nav channels can resume generating action potentials (Fig. 4C). We used a peak current of 96 pA for the strong stimulus, which is a rather conservative magnitude considering odor-evoked receptor currents in rodents have been reported of >200 pA (*43*, *45*–*47*). Together, these data suggest that the shift in perception occurring at higher concentrations, as depicted in Fig. 1B, is a result of action potential failure within the primary sensory neurons situated in the nasal epithelium.

### Learning perceptual constancy involves peripheral changes

**Fig. 5.**
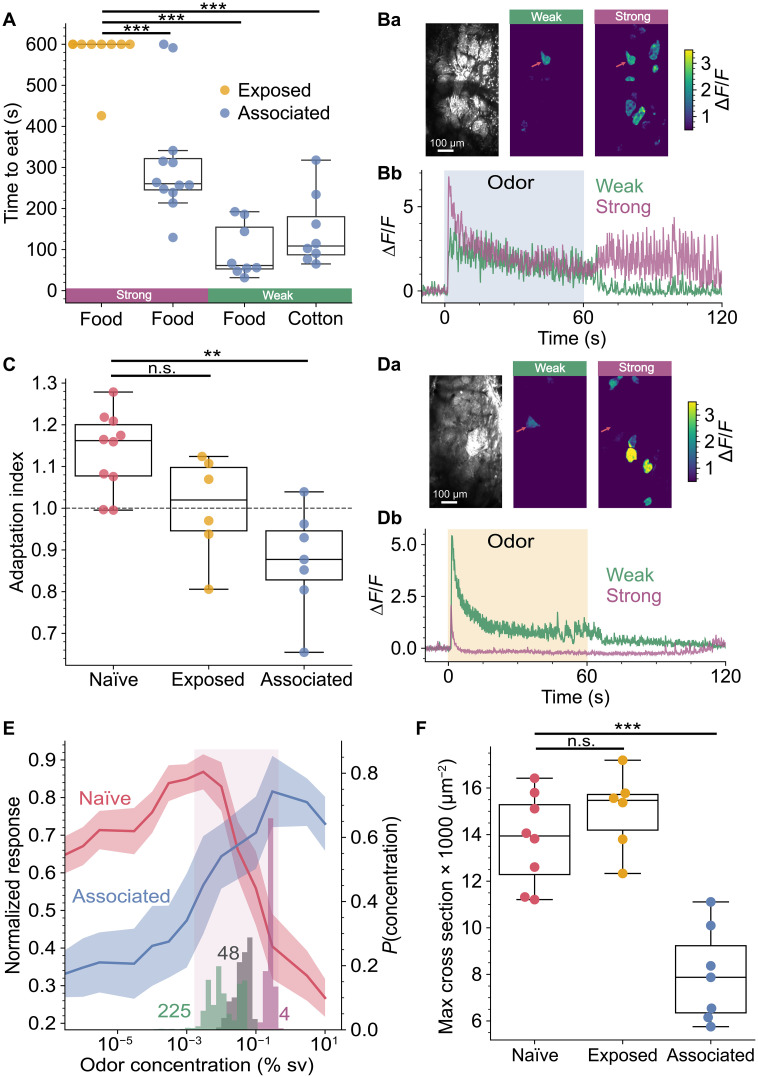
Learning perceptual constancy. (**A**) The latency to eating for cohorts of mice fed 2.5% ethyl tiglate–scented food for 1 week (blue dots) was significantly lower than mice exposed to the same concentration (yellow dots; *P* = 4.0 × 10^−6^, Kruskal-Wallis). Mice were tested with a buried food pellet of the same concentration (purple bar; *P* = 0.0018, corrected Mann-Whitney *U*) or with a 1 × 10^−3^% food pellet (green bar food; *P* = 0.0012) or cotton ball (green bar cotton; *P* = 0.0012). (**Ba**) Field of view and response maps for weak percept and strong percept (1 × 10^−3^ and 3%, respectively) from a mouse after associating ethyl tiglate with food. Note the primary glomerulus is still evident in the strong percept. (**Bb**) Time courses of the responses in (Ba). (**C**) There was a significant difference between the adaptation indices of naïve, associated, and exposed mice [*P* = 4.7 × 10^−4^, one-way analysis of variance (ANOVA)]; the associated cohort was significantly lower than naïve (*P* = 3.3 × 10^−4^, Tukey), whereas mice exposed to the same concentration were not significantly different (*P* = 0.076), all measured at the strong percept. (**D**) Same as (B), but for mice that have been exposed to ethyl tiglate without food association. (**E**) Normalized concentration response curves for the primary glomerulus displayed as mean ± SEM for the naïve and associated cohorts. Right axis: Probability distributions of the concentrations of the ethyl tiglate-scented food measured at distances of 4, 48, and 225 mm within the home cage. Shaded area shows the range of concentrations overlapping with the steepest part of the concentration response curve for associated mice. (**F**) There was a significant difference in the size of the primary glomerulus (*P* = 5 × 10^−6^, one-way ANOVA); it was significantly smaller in mice that had associated ethyl tiglate with food compared to naïve mice (*P* = 4.3 × 10^−5^, Tukey), whereas those exposed to ethyl tiglate were not significantly different (*P* = 0.49, Tukey).

We have described the failure of perceptual constancy for an unfamiliar odor and its underlying mechanism. The inability to recognize the same object across different concentrations would clearly be disadvantageous, particularly for salient odors such as food. Natural interaction with food stuffs would enable an animal to associate a range of concentrations with the same object; consuming the food provides much weaker activation of the olfactory epithelium by retronasal olfaction (*48*). We therefore investigated whether natural ‘passive’ association of the odor with food was able to endow perceptual constancy for ethyl tiglate across the full range of concentrations we used. We provided ethyl tiglate mixed with standard chow as a food source at a concentration corresponding to the strong percept (2.5%). After 1 week of exclusively consuming ethyl tiglate–scented food, we performed food finding tests. Mice, after an overnight fast, were placed in a cage with a buried food pellet, scented with 2.5% ethyl tiglate. Mice that had associated ethyl tiglate with food found the food pellet faster than a cohort of mice that had experienced the same amount of ethyl tiglate over the preceding 7 days but not associated with food (154 ± 96 versus 332 ± 122 s; *P* = 0.036). The mice had clearly formed an association of ethyl tiglate with food as 11 of the 12 mice tested also began eating the pellet within the 10-min test, whereas only 1 of the 9 “exposed” mice did so (Fig. 5A). These data indicate that the mice have formed an association of ethyl tiglate at the strong percept with food; we next sought to test whether this association extended to weaker concentrations that, in naïve mice, correspond to a different weak percept. Mice fed 2.5% ethyl tiglate were tested with a buried food pellet scented with 1 × 10^−3^%. All mice rapidly found the pellet and began eating (Fig. 5A). We were concerned that, at this lower concentration, the smell of the standard chow may be aiding their localization of the food, so we also performed the test with a buried cotton ball soaked in the same weak concentration. Unexpectedly, all mice rapidly found and began nibbling the cotton ball (Fig. 5A and movie S1) and this latency was not different to that for the food pellet scented with the same odor (*P* = 0.64 corrected Mann-Whitney *U*). These data indicate that, after consuming a strong concentration of the odor, mice associate a broad range of concentrations of the odor with food, even concentrations that previously evoked a different percept. We next asked what neural changes underpinned this learning-induced change in perception; would the sensitive primary glomerulus alter its properties to maintain responsiveness across the concentration range and/or shift to always being the first glomerulus to activate? Again, we could easily identify the same primary glomerulus; it was the only one active at weak concentrations (Fig. 5Ba). When we generated response maps similar to Figs. 2A and 3C, the primary glomerulus was obvious at both the weak and strong concentrations (Fig. 5B). This was due to the primary glomerulus responding throughout the whole stimulus period (Fig. 5Bb), in stark contrast to what is observed in naïve mice (Figs. 2H and 3D). This is quantified in Fig. 5C, which shows that the amount of adaptation in the primary glomerulus is much lower in mice that have associated ethyl tiglate with food (AI = 0.87 ± 0.047, *N* = 6) compared to naïve mice (AI = 1.14 ± 0.028). We found the same effect when we measured the signal from the presynaptic terminals of the primary glomerulus in OMPxGCaMP6f mice; the AI index was significantly smaller in the 2 mice that had associated ethyl tiglate with food compared to 13 naïve mice (*P* = 3.9 × 10^−4^, independent *t* test). We also examined the neural activity in mice that were exposed to the same amount of ethyl tiglate but not associated with food. These exposed mice still displayed close to complete adaptation in the primary glomerulus with a mean AI of 1.002 ± 0.045 across the six animals tested (Fig. 5, C and D), which was not significantly different to the naïve animals. A shift from complete adaptation, caused by depolarizing block, to a sustained response implies a shift in sensitivity of the primary glomerulus. When we plotted the magnitude of response (calculated as the integral over the stimulus period) as a function of concentration, a marked shift in sensitivity was observed between naïve animals and those that associated ethyl tiglate with food (Fig. 5E); those only exposed to ethyl tiglate were intermediate between the two (fig. S1). The concentration at which the maximum response to ethyl tiglate occurred shifted by approximately two orders of magnitude, aligning closely with the concentration present in the food. We also found that the dynamic range of the primary glomerulus became more closely aligned with the range of concentrations that would be experienced within the home cage (Fig. 5E) yet maintained sensitivity to very low concentrations. Mice that had formed a food-odor association investigated at every concentration in the cross-habituation test of Fig. 1 (fig. S2).

We expected that, after food association, the primary glomerulus would shift to being the first to activate across all concentrations; however, it continued to lag other glomeruli at concentrations corresponding to the strong percept (fig. S3). This suggests that the primacy (relative activation time) of the glomerular input appears to be unalterable, at least with natural interaction with an odor object. Fear association has demonstrated effects on olfactory receptor neurons, which results in altered glomerular size (*49*, *50*). We also found that, in mice that had formed an association of ethyl tiglate with food, the size of the primary glomerulus was smaller than in naïve mice (Fig. 5F), reminiscent of the structural changes observed with fear conditioning (*49*, *50*) but of the opposite sign.

**Fig. 6.**
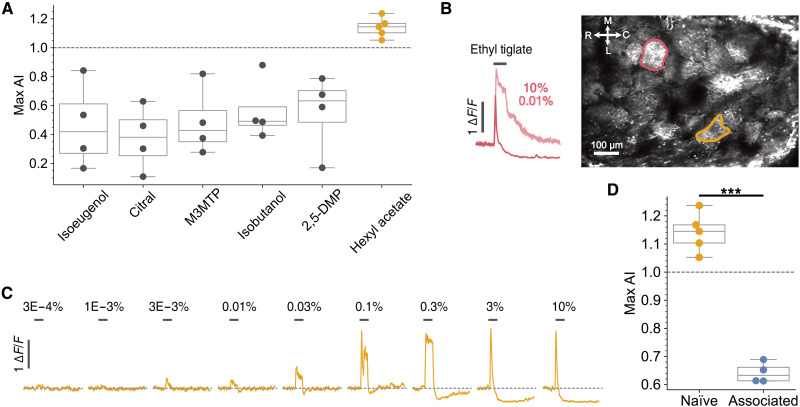
Rapid adaptation phenotype is odor specific. (**A**) Maximum AI detected across all glomeruli within a field of view in response to 3-s presentations of six different odors at 10%; each dot represents an individual mouse [*N* = 4 for isoeugenol, citral, methyl 3-(methylthio)propionate (M3MTP), isobutanol, and 2,5-dimethylpyrazine (2,5-DMP); *N* = 5 for hexyl acetate]. (**B**) Example field of view in a Pcdh21xGCaMP6f mouse highlighting the typical location of glomeruli that undergo rapid adaptation in response to ethyl tiglate (red) and hexyl acetate (yellow). Inset shows time course for 3-s presentations of ethyl tiglate at 0.01 and 10%. M, medial; C, caudal; L, lateral; R, rostral. (**C**) Time course for yellow glomerulus highlighted in (B) in response to 3-s presentations of hexyl acetate, indicated by gray bars. (**D**) The maximum AI detected across all glomeruli within a field of view in response to 3-s presentations of hexyl acetate at 10% was significantly lower in mice that were fed 2.5% hexyl acetate mixed in their normal diet for 1 week (blue dots; *N* = 4) than for naïve mice (yellow; *N* = 5; *P* = 3.83 × 10^−5^, unpaired *t* test), with each dot representing an individual mouse.

We next sought to test whether high concentrations of other odors result in transmission failure to glomeruli similar to Figs. 2 to 4 and whether this is also modulated by learning as in Fig. 5. We used six odors at 10% to test whether these evoked glomerular responses with an AI of ≥1, a hallmark of transmission failure. Figure 6A summarizes these results by showing the highest AI measured across all active glomeruli at 10% for each odor. Of the six odors tested, only hexyl acetate generated a response with an adaption index greater than 1. Our imaging area was restricted to the dorsal surface, so it is possible that, for the other five odors, glomeruli located elsewhere on the olfactory bulb generated responses with adaptation indices greater than 1. Alternatively, some odors may lack sufficient efficacy to generate receptor currents large enough to cause depolarizing block and transmission failure. The maximal response of olfactory receptor neurons is odor dependent (*51*). Nevertheless, hexyl acetate did generate a response dynamic typical of depolarizing block (*N* = 5) and this was located more caudally than the primary glomerulus for ethyl tiglate, in the three mice where both odors were tested (Fig. 6, B and C). Similar to Fig. 2, the hexyl acetate glomerulus shifted from sustained responses for weaker concentrations up to 0.3% (AI = 0.54 ± 0.034, *N* = 5) to a transient response that rapidly dropped below baseline with an AI of 1.15 ± 0.031 (*N* = 5), although this glomerulus only began responding to hexyl acetate at ~0.01% (Fig. 6C). We showed that associating ethyl tiglate with food resulted in changes that prevented transmission failure, preventing the response from dropping below baseline (Fig. 5). To test whether the hexyl acetate-adapting glomerulus is also subject to such plasticity, we fed a cohort of mice chow scented with 2.5% hexyl acetate for 1 week. As we were not confident in identifying the same glomerulus across animals for this odor, we simply compared the maximum AI evoked by 10% hexyl acetate across all glomeruli. We found a significant reduction in the AI (Fig. 6D) switching from 1.15 ± 0.031 (*N* = 5) in naïve mice to 0.64 ± 0.018 (*N* = 4) in mice that had been fed hexyl acetate (*P* = 3.83 × 10^−5^). Overall, these data indicate that, when mice make an association of an odor with food, changes must occur within olfactory receptor neurons that match their sensitivity to the odor, preventing transmission failure. This could be due to changes in the receptor compliment (*52*) or alterations of intrinsic membrane properties (*53*). Such range matching likely aids food localization as, with neural responses more sensitive to relevant concentrations, mice will be better able to follow a concentration gradient.

## DISCUSSION

We show that mice can experience a concentration-induced shift in odor perception (Fig. 1), similar to reports in humans (*54*, *55*). This shift in perception coincides with a failure in transmission from receptor neurons to a single primary glomerulus in the olfactory bulb (Figs. 2 to 4). After association of the odor with food, such transmission failure is prevented and a single percept exists for a broad range of concentrations (Fig. 5). These data are consistent with odor identity relying on a sparse code. Previous works have also suggested a sparse identity code based on the relative activation times of different glomeruli, with those activating earliest carrying more information (*25*, *26*). At weaker concentrations, and for naïve odors, our data are consistent with this model; the primary glomerulus activates first at weaker concentrations, but at intensities perceived as a distinct percept, it is no longer first (Figs. 1B and 2F). However, after learning, when a broad range of concentrations evoke the same “food” percept, the primary glomerulus still lags that of others at high concentrations (fig. S3), despite obvious shifts in its sensitivity (Fig. 5). It may be that the nature of the task influences the coding strategy used. When mice are trained in operant discrimination tasks, where reward is contingent upon a prompt behavioral action, mice learn to make discrimination decisions quickly (*25*, *26*), albeit after a lot of training. This is markedly different to what occurs with natural interaction with an odor object. In our experiments, mice passively interact with odorized food and, by doing so, will experience a range of concentrations of the odor that they will correlate with their distance to the object (*56*). In such a scenario, the activity of the most sensitive glomerulus will show the highest degree of covariance with the odor stimuli, for example, it will be the only one activate at larger distances from the object. Over repeated interactions, this primary glomerulus would therefore be given the most weight in determining the presence of the object. This idea of coherent covariation is used to explain the acquisition of semantic concepts (*57*) and would naturally give rise to a sparse odor identity code, especially with the observation that, at low concentrations, a sparse and structured representation of chemical space exists in glomerular activity (*22*). Such sparse codes for monomolecular odors are likely well suited to encoding the more complex mixtures found in natural odors, achieved by linearly combining the sparse representations of their individual constituents (*58*).

Our data indicate that the rapid adaptation observed at higher concentrations of ethyl tiglate and hexyl acetate in naïve mice is likely due to action potential failure within the olfactory receptor neuron (Figs. 3 and 4); such a behavior at higher odor concentrations is evident in many recordings from olfactory receptor neurons (*44*, *46*, *59*) and has also been demonstrated in Drosophila larvae (*60*). This failure of transmission occurs due to a mismatch between membrane resistance and receptor currents; olfactory receptor neurons have very high input resistances of ~4 to 5 gigohms (*45*), whereas odor-evoked currents in these cells can be as large as 200 pA (*43*, *45*–*47*). With a simple-minded “ohmic” calculation, such receptor currents would cause an 800- to 1000-mV depolarization. Of course, the receptor current is not an ideal current source; rather, it has a reversal potential, dominated by the Ca^2+^-activated Cl^−^ current ANO2 (*43*, *61*). Thus, rather than an 800-mV depolarization, the membrane will become clamped at a depolarized potential. This sustained depolarization locks Nav channels in their inactivated state, preventing transmission of action potentials down the axon. We do not yet know whether this behavior is typical for every odor, although it is evident at higher odor concentrations in many recordings from olfactory receptor neurons (*44*, *46*, *59*) and has also been demonstrated in Drosophila larvae (*60*). Figure 6 demonstrates that some odors do not generate depolarizing block. However, the absence of evidence does not necessarily imply evidence of absence. Our recordings were limited to the dorsal surface of the olfactory bulb, so it may be that glomeruli located outside of our field of view do generate rapid adaptation at high concentrations; we were just unable to measure their responses. Nevertheless, several factors may determine whether an odor is capable of inducing depolarizing block. Odorant-receptor interactions are described by two properties: affinity describes sensitivity, whereas efficacy determines the maximal response that can be generated. Many odors act as partial agonists (submaximal efficacy) (*51*, *62*) and are therefore unable to generate the largest receptor currents, which can induce depolarizing block. The concentration achieved at the receptor binding site is also influenced by the volatility and hydrophobicity of each odor (*63*). Our data also show that previous interaction with an odor can prevent rapid adaptation indicative of depolarizing block (Figs. 5 and 6). Multiple factors may therefore determine whether high concentrations of an odor result in transmission failure, which we have linked to a perceptual shift in odor quality. Concentration-dependent shifts in odor perception have also been described in human olfactory experiments (*54*, *55*) although likely less than 10% of odors do so (*64*). The apparent low incidence of such perceptual shifts in humans is likely due to a combination of odorant/receptor properties and previous experience. Object constancy in vision is not fully developed in human infants (*1*, *2*); perhaps, odors that generate concentration-dependent percepts are more prevalent in human infants that have had less experience to shape how their brain responds to their environment.

Depolarizing block may seem like a flaw in how the olfactory system operates, unless one considers that the primary goal of the olfactory system is first to detect odors and then to classify them. After exposure to a salient odor, the olfactory receptor neurons adjust their sensitivity, so that their maximum response falls near the concentration of the salient object and transmission failure no longer occurs at high intensities, supporting perceptual stability (Fig. 5). We also find that the size of the glomerulus is reduced after associative learning, implying that there may be a reduction in the number of olfactory receptor neurons projecting to the primary glomerulus or remodeling of mitral/tufted dendrites. Such plasticity within the nose bears resemblance to aversive conditioning, whereby an odor paired with a foot shock brings about increased glomerular input for the conditioned odor by generating more olfactory receptor neurons carrying that receptor (*49*, *50*), whereas subsequent extinction reverses this effect (*65*). Such changes only occurred when the odor was given salience; they did not occur with exposure without fear conditioning; similarly, we find that only association with food causes a change in glomerular size. It seems then that the nasal epithelium is a particularly dynamic structure able to tailor cell generation and receptor densities to optimally encode salient features encountered in the environment.

## MATERIALS AND METHODS

### Animals

Animal handling and experimentation were carried out according to the UK Home Office guidelines and the requirements of the United Kingdom (Scientific Procedures) Act 1986 and the University of Leeds animal welfare ethical review board (under license PBA51A138). Mice were housed under a 12-hour:12-hour light:dark cycle with free access to food and water. All efforts were made to minimize animal suffering and the number of animals used. Pcdh21-nCre mice [C57BL/6Cr-Tg(Pcdh21-cre)BYoko (RBRC02189)] and OMP-Cre mice [B6;129P2(Cg)-Omp<tm4(cre)Mom>/MomTyagRbrc (RBRC02138)] were crossed with floxed GCaMP6f mice [GCaMP6f.flox, stock 028,865, B6J.CgGt(ROSA)26Sor<tm95.1 (CAGGaMP6f)] to generate Pcdh21xGCaMP6f mice and OMPxGCaMP6f mice, respectively. Pcdh21-nCre and OMP-Cre mouse lines were originally obtained from RIKEN BioResource Research Center (Ibaraki, Japan), with permission from P. Mombaerts, the original developer of the OMP-cre line (*30*, *31*). The GCaMP6f mouse line was obtained from the Jackson Laboratory (Maine, United States). All mouse lines were maintained in house. Consistent with the NC3Rs guidelines (https://nc3rs.org.uk/who-we-are/3rs), both males and females aged 2 to 4 months old were used in this study.

### Odor stimuli

Odorants were obtained from Sigma-Aldrich or Alfa Aesar. Liquid dilutions of odorants were prepared to achieve desired concentrations of ~3 × 10^−5^, 1 × 10^−4^, 3 × 10^−3^, 1 × 10^−2^, 0.1, 1, 3, and 100% using serial dilutions. Odorants were diluted in oil either (Sigma-Aldrich, 69794) or (Spectrum Chemical, C3465) within ~1 week of experiments. Diluted odorants were delivered in vapor phase in synthetic medical air using either an 8- or 16-channel olfactometer (Aurora Scientific, 206A or 220A, respectively). Total flow rates from the olfactometers were kept constant at 1000 sccm (standard cubic centimeter per minute). In imaging experiments, the output tubing of the olfactometer was positioned 1 to 2 cm in front of the mouse’s nose. Odorant presentations were always delivered in increasing concentrations. Interstimulus intervals were extended as the odor concentration increased, varying between 20 and 120 s to minimize any adaptation. All odor concentrations are reported as % saturated vapor (sv). Odor concentrations delivered to the behavior boxes used in Fig. 1 were measured with a miniPID (Aurora Scientific, 200B) placed at the nose port and are reported relative to the % sv used for imaging experiments. The odor concentrations experienced in the home cage with odorized food, reported in Fig. 5E, were also measured with a miniPID placed in the home cage at different distances from the food. Distributions of the concentration are shown measured over a 490-s window and are reported relative to the % sv used for imaging experiments.

### Behavior

#### Cross-habituation test

Cross-habituation experiments were set up similar to the method described in (*10*). Two- to 3-month-old mice were placed in a 25 cm–by–25 cm perspex chamber with all sides opaque. Each chamber was fitted with an odor port and exhaust tube at opposing sides. The output of the olfactometer was connected to the odor ports of four chambers using identical path lengths of Teflon tubing; the flow rate from the olfactometer was 1000 sccm. There was no difference in the concentration of odor delivered to each box as measured with a minPID. Each odor port housed an infrared beam break sensor (The Pi Hut). Beam break events and valve openings were logged using a MicroPython pyboard lite (v1.0) and pyControl GUI (v1.6). A mini vacuum pump (SLS2602) was attached to the exhaust tubes of all four chambers via tubing with an identical path length, and air was extracted at a rate of 5.5 liters/min. In each trial, mice were presented with either oil or a test odor for 60 s, followed by 60 s of synthetic medical air. Wild-type C57BL/6 mice were first habituated to the test environment for 10 min, before starting the stimulus protocol shown in Fig. 1Ac. Each presentation lasted 60 s with 60 s of medical air between presentations. In all instances, animals were naïve to the stimuli. Each animal was tested with 2-heptanone and ethyl tiglate with 1 day between experiments, with half the cohort tested with ethyl tiglate first and the other half with 2-heptanone.

#### Head-fixed perception test

Wild-type C57BL/6 mice were anesthetized with isoflurane on a custom stereotaxic frame for head-bar attachment. Anesthesia was maintained at a level of ~1.5 to 2% isoflurane, 1 liter/min O_2_ during surgery. Metacam (5 mg/kg, subcutaneously) and buprenorphine (0.1 mg/kg, intraperitoneally) were administered as analgesics. A small piece of skin above the skull, big enough for placing the head bar, was carefully removed and cleaned with a sterile saline solution. Superglue was initially applied over the exposed skull followed by dental cement to affix a custom 3D printed head bar. Additional dental cement was applied to cover the head bar and the exposed skull. Postsurgery mice were given soaked diet and buprenorphine (0.1 mg/kg, intraperitoneally) for the following 2 days; all mice were allowed 1 week for recovery before habituation to head-fixation. Mice were handled 5 min each day for 2 days prior to behavioral tests, aiming to acclimate them to the experimenter. Mice were head-fixed on a treadmill, described in (*13*), and habituated for 10 to 20 min per day for 2 to 3 days before recordings. The mouse face was imaged with a Basler camera (catalog no. 107652) with 12-mm Edmund Optics lens (catalog no. 33-303), and videos were captured at 120 Hz with 750-nm illumination (outside the visual range of mice). Odors were delivered using an olfactometer (220A, Aurora Scientific) and custom-written code. The recording and synchronization of data was performed with Bonsai-Rx (*66*) and a Teensy 4.2 microcontroller (PJRC). Each video acquisition was 35 s, composed of 10 s of baseline, 10-s stimulus, and 15-s poststimulus. Each mouse was first presented with five to seven oil trials before the odor, and all trials were spaced ≥60 s apart. A DeepLabCut (*14*) neural net was trained on 15 frames from each mouse and used to extract the *xy* coordinates of the key points from every frame.

#### Passive odor association

The diets of mice were supplemented with ethyl tiglate for 7 days. Ethyl tiglate was diluted in distilled water (1:40), before being combined with their regular diet in powdered form (equal w/v) and shaped into a single ball (~5 g per mouse). Each mouse received a fresh food ball daily at ~17:30 in a glass dish (7.5 cm wide, 4.25 cm deep).

#### Perceptual odor exposure

The environments of wild-type mice were enriched with ethyl tiglate for 7 days. Ethyl tiglate was diluted in oil (1:40) and 1 ml was applied to Whatman filter paper. Odorized filter paper was folded inside a metal tea ball and placed inside the animal’s home cage, replenished daily at ~17:30.

#### Food and odor finding test tests

Wild-type mice were fasted for ~16 hours before testing commenced. A clean housing cage was filled with ~4 cm of fresh bedding, and an odorized food or cotton ball (~1.5 cm^3^) was hidden beneath the bedding in a single corner. Care was taken not to leave odor trails during food/cotton ball placement. Mice were placed in the cage and a timer was set once a clear perspex lid had been attached. The time taken for mice to locate (defined as when most of the food/cotton ball became visible) and start eating the food/cotton ball was manually recorded.

### In vivo two-photon Ca^2+^ imaging

Mice were anesthetized with urethane (1.5 g/kg), and body temperature was maintained at 37°C. Animals were secured with a custom-made head bar, and a craniotomy covering the right hemisphere of the olfactory bulb was performed. The exposed bulb was covered with 2% low–melting point agarose in artificial cerebrospinal fluid, and a 3-mm glass coverslip (Biochrom) was affixed with dental cement. Silicone rubber (Body Double Fast Set) was applied to the skull surrounding the cranial window to create a well for the water dipping objective of the microscope. For experiments where drugs were topically applied, segments of dura were removed and the animal was imaged without a coverslip. GCaMP6f fluorescence was imaged with a custom-built microscope, excited at 940 nm using a pulsed Mai Tai eHP DeepSee TI:Sapphire laser system (SpectraPhysics). A resonant-galvo mirror assembly (Sutter Instruments) scanned the beam through a 16× water-dipping objective (N16XLWD-PF, numerical aperture: 0.8, Nikon). Fluorescence was detected using GaAsP photomultiplier tubes and appropriate filters and dichroic mirrors. Images were acquired at 30 to 120 Hz, using ScanImage software (*67*).

### Pharmacology

The GABA_B_ receptor antagonist CGP 54626 hydrochloride (Tocris Bioscience) was used at a concentration of 5 μM. The dopamine D2 receptor antagonist raclopride (Tocris Bioscience) was used at a concentration of 100 μM. Both drugs were dissolved in artificial cerebrospinal fluid (7.4 pH, 135 mM NaCl, 5.4 mM KCl, 5 mM Hepes, and 1.8 mM CaCl_2_·2H_2_O) and topically applied to the olfactory bulb 20 min before imaging recommenced.

### Data analysis

#### Image segmentation of glomeruli

The Suite2p pipeline v0.10.1 (*68*) was used to register data with the default options (“nimg_init”: 300, “batch_size”: 500, “maxregshift”: 0.1, and “smooth_sigma”: 1.15); regions of interest corresponding to glomeruli were manually drawn in FIJI (*69*), and raw fluorescence was extracted from glomeruli using a custom-written code in Python. Extracted fluorescent traces were normalized as Δ*F*/*F* using the following equation: *F* − *F*_0_/*F*_0_, where *F* is the raw fluorescent trace and *F*_0_ is the baseline fluorescence recorded 5 s prior to odor stimuli.

#### Adaptation index

To quantify the amount of adaptation, we defined the AI as the difference between the peak response (A in Fig. 2G) and the mean of the last 100 ms of the stimulus period divided by the peak response. Before calculating AI, data were filtered with a five-point mean filter.

#### Response maps

For each stimulus, response maps were generated using the following equation: *F* − *F*_0_/*F*_0_, where *F* is the raw fluorescent movie and *F*_0_ is the 2D image of mean fluorescence recorded 3 s preceding the odor stimulus. Maps are displayed after two-dimensional Gaussian filtering with a sigma of 2, and areas outside the segmented glomeruli were set to zero.

#### Classifier

We calculated the responses for each glomerulus by taking the mean of the Ca^2+^ signal over the stimulus period and 1 s after odor cessation (accounting for delayed activation seen in some glomeruli). Glomeruli were only considered to be responsive if their signal-to-noise ratio was ≥5, defined as: (max amplitude over stimulus window − mean amplitude over 3 s preceding stimulus)/SD over 3 s preceding stimulus, and that successive concentrations of the same odor were responsive. Trials where irregular breathing was apparent were excluded, i.e., a drop in activity across all glomeruli. One to three trials of each odor concentration were delivered, and responses were normalized to the maximum response across all stimuli. Odor responses were assigned a percept label if they were within 50% of the boundary concentration shown in Fig. 1B. These data were classified using a linear support vector machine (class weight = balanced) from the scikit-learn library. The classifier accuracy was evaluated using the Leave-One-Out cross-validator to calculate weighted average F1 scores as reported in Figs. 2, B and D. Relative glomerular weighting (Fig. 2C) was obtained by calculating the absolute values of the coefficients for each glomerulus and normalizing each value to the largest assigned weight.

#### Ranking glomerular activation times

To determine the first active glomerulus for a given stimulus, we identified the first frame during the stimulus period with a signal-to-noise ratio ≥5. The time stamp of this initial frame was taken as the activation time for the glomerulus. Trials where irregular breathing was apparent were excluded. For each stimulus, all responsive glomeruli were assigned a rank, with the first active glomerulus assigned a rank of 1. For trials with multiple repeats, the primary glomerulus was assigned the mean rank it received across all trials of the same concentration.

#### Glomerular size measurements

To determine the maximal cross-sectional area of primary glomeruli, we obtained a *z*-stack at 2- to 4-μm increments of the olfactory bulb in each mouse. Glomerular outlines were manually drawn in Fiji (*69*) at each plane with the largest taken as the maximal cross-sectional area. These results were confirmed by a labeler blind to the groups. Where glomerulus structure was difficult to determine in stacked images alone, outlines were drawn using odor-evoked recordings.

#### Statistical analysis

For all statistical parameters, data were first tested for normality with Shapiro-Wilk and are reported as mean ± SEM if normal or median ± median absolute deviation if not. Paired comparisons used a *t* test or Wilcoxon signed-rank test as appropriate. The data in Fig. 1B were tested with a Friedman test comparing the median of the last five oil presentations with each of the odor deliveries; Wilcoxon signed-rank test was used for post hoc comparisons with Bonferroni correction. All *P* values were adjusted for multiple comparisons with Bonferroni correction. Where asterisks are used to indicate significance, *P < 0.05, **P < 0.01, and ***P < 0.001. In all cases, *N* refers to the number of animals.

### Model

Morphologically realistic models of olfactory receptor neurons and their receptor input were simulated in NEURON 8.2 (*70*). Each OSN consisted of four compartments: an axon of length 1.6 mm and diameter of 0.2 μm (*71*), a soma with diameter of 5 μm, a dendrite with a length of 12 μm and 0.8 μm in diameter, and an end bulb of 2 μm in diameter, as shown in Fig. 4A. Axial resistance was 180 ohm·cm, and membrane capacitance was 1 μF cm^−2^. Standard Hodgkin-Huxley channels were used at a uniform density throughout the cell with the following conductance densities: Na = 32 mS cm^−2^, K = 8 mS cm^−2^, passive = 0.08 mS cm^−2^, and passive reversal = −50 mV. This gave an input resistance of 4 gigohms similar to the reported membrane resistance of OSNs (*45*). To mimic the basal firing activity of olfactory receptor neurons evoked by spontaneous Nav channel openings in the end bulb (*72*), Gaussian noise with a mean of 0.5 pA and SD of 0.014 was injected into the end bulb compartment, which generated spontaneous firing at ~7 Hz similar to the reported spontaneous rates (*45*).

The receptor currents were modeled as a point process placed on the tip of the end bulb with a time course described by three piecewise functions obtained from fits to the synaptic currents reported in (*44*), where adaptation to sustained odor stimuli was directly measured by using sustained odor pulses. The three piecewise functions correspond to the onset and duration of the odor stimulus (*a*), the decay after the stimulus (*b*), and the adaptation during the steady-state phase of the stimulus (*c*). The synaptic conductance (*g*) was therefore g=m(a+b−c), where *m* is a scaling factor. For the weak odor concentration:

$$a\left(t\right)=\left\{\begin{array}{cc} \frac{0.0415}{1+e\left(\frac{190+t_{0}-t}{40}\right)} & \text{if}\;t_{0}<t<t_{0}+t_{d}\times t_{x}\\ 0 & \text{otherwise} \end{array}\right.$$

$$b\left(t\right)=\left\{\begin{array}{cc} \frac{0.017}{1+e\left(\frac{-200.55+t_{0}+t-t_{d}\times t_{x}}{100.14}\right)} & \text{if}\;t_{0}+t_{d}\times t_{x}<t<t_{0}+t_{d}\times t_{x}+1500\\ 0 & \text{otherwise} \end{array}\right.$$

$$c\left(t\right)=\left\{\begin{array}{cc} 0.041-0.010774+0.03674e\left(\frac{-t+t_{d}+190}{1232.7}\right) & \text{if}\;t_{0}+190<t<t_{0}+t_{d}\times t_{x}\\ 0 & \text{otherwise} \end{array}\right.$$

For the strong odor concentration:

$$a\left(t\right)=\left\{\begin{array}{cc} \frac{0.08}{1+e\left(\frac{90+t_{0}-t}{20}\right)} & \text{if}\;t_{0}<t<t_{0}+t_{d}\times t_{x}\\ 0 & \text{otherwise} \end{array}\right.$$

$$b\left(t\right)=\left\{\begin{array}{cc} \frac{0.0482}{1+e\left(\frac{-606.55+t_{0}+t-t_{d}\times t_{x}}{240.14}\right)} & \text{if}\;t_{0}+t_{d}\times t_{x}<t<t_{0}+t_{d}\times t_{x}+2500\\ 0 & \text{otherwise} \end{array}\right.$$

$$c\left(t\right)=\left\{\begin{array}{cc} 0.08-0.04474+0.03674e\left(\frac{-t+t_{d}+190}{454.54}\right) & \text{if}\;t_{0}+190<t<t_{0}+t_{d}\times t_{x}\\ 0 & \text{otherwise} \end{array}\right.$$

Where *t*_0_ is the odor stimulus onset in milliseconds, *t_d_* is the stimulus duration in milliseconds, and *t_x_* is a duration multiplier to reflect that the receptor current outlasts the stimulus with this duration increasing with both the intensity and duration of the stimulus (*42*–*44*, *46*). For the weak stimulus, *t_x_* was set at 1 and for the strong stimulus *t_x_* was 1.65 + a value drawn at random from a Gaussian distribution with a mean of 0.2 and SD of 0.25 to reflect heterogeneity in the response decay across neurons carrying the same receptor (*73*). Peristimulus time histograms were computed for 500 olfactory receptor neurons at each concentration with bin widths of 50 ms as shown in Fig. 4E. To estimate the Ca^2+^ signal that GCaMP6f would report for each odor concentration, the mean spike rate was convolved with a kernel representing the kinetics of GCaMP6f (*18*) defined by

$$g\times \frac{t}{0.001}\times \text{exp}\left(-\frac{t+0.15}{0.15}\right)$$

where *g* is a scaling factor. In our model, action potentials are initiated virtually simultaneously in the end bulb and soma owing to the high membrane resistance giving a length constant of ~740 μm.
