# Supplementary material for: Perceptual constancy for an odor is acquired through changes in primary sensory neurons
Source: Sci Adv. 2024 Dec 11;10(50):eado9205. doi: 10.1126/sciadv.ado9205 (PMC11633753; doi:10.1126/sciadv.ado9205)
Supplement: Supplementary file 1 — Figs. S1 to S3 Legend for movie S1 [file sciadv.ado9205_sm.pdf]

Science Advances
AAAS

Supplementary Materials for

Perceptual constancy for an odor is acquired through changes in primary sensory neurons

Mark Conway *et al.*

Corresponding author: Jamie Johnston, j.johnston@leeds.ac.uk

Sci. Adv. **10**, eado9205 (2024)
DOI: 10.1126/sciadv.ado9205

The PDF file includes:

Figs. S1 to S3
Legend for movie S1

Other Supplementary Material for this manuscript includes the following:

Movie S1

Supplemental Figures

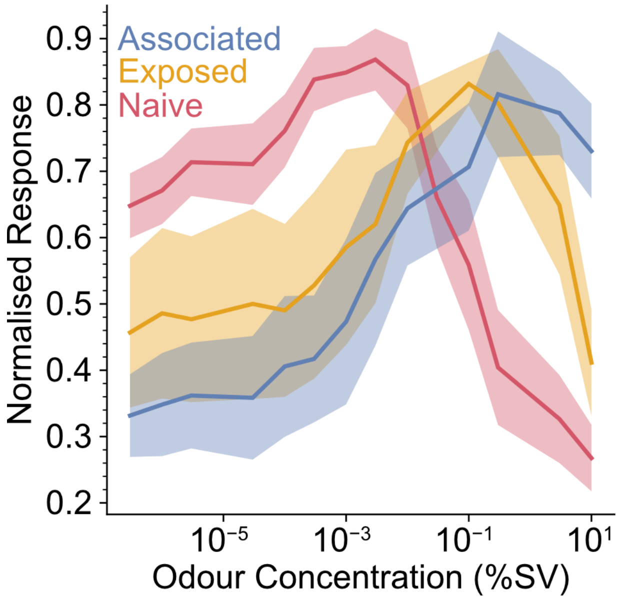

Figure S1 Sensitivity of naïve, exposed and associated cohorts. Normalised concentration response curves for the primary glomerulus displayed as mean ±SEM for the naïve (N = 10), exposed (N = 6) and associated (N = 7) cohorts.

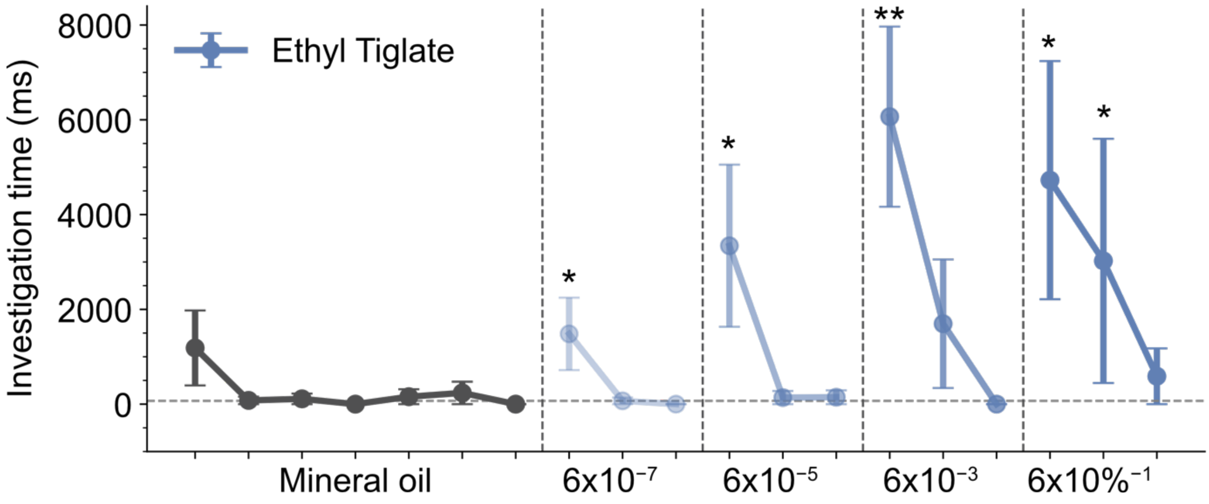

Figure S2 Cross-habituation of ethyl tiglate concentrations after associating ethyl tiglate with food. Odour investigation times during stimulus delivery of ethyl-tiglate for mice that had been fed 2.5% ethyl tiglate scented food for 7 days. Data are displayed as median ± the median absolute deviation, N=16. The horizontal dashed line indicates the basal amount of investigation calculated from the last 5 oil presentations. There were significant differences between the last 5 oil presentations and presentations of ethyl-tiglate ($p = 6.02 \times 10^{-18}$, Friedman test). Asterisks indicate post-hoc significance tests above the basal investigation (see methods).

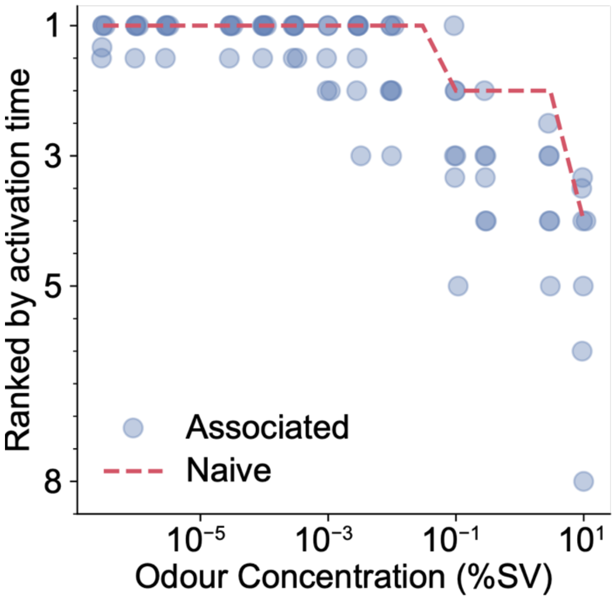

Figure S3. No change in activation rank of the primary glomerulus after associating ethyl tiglate with food. The activation rank of the primary glomerulus as a function of concentration, each mouse represented by a dot (N=7), the dashed red line indicates the median from naïve mice (N=9).

Movie S1. Finding ethyl tiglate soaked cotton ball. A mouse that had been fed 2.5 % ethyl tiglate for 1 week at the end of a food finding test where a cotton ball soaked with $1x10^{-3}$ % ethyl tiglate had just been uncovered. The mouse nibbles the cotton ball.
